# The Microscope in Medicine and Dentistry

**Published:** 1873-02

**Authors:** D. C. Hawxhurst


### The Microscope in Medicine and Dentistry.

BY D. C. HAWXHURST.

The microscope is just now attracting universal attention. Every earnest student of life in the department of either physiology or pathology, is at this time looking with peculiar interest to its revelations. The European press teems with the results of microscopic research. Our own publishers are beginning to give us books and periodicals on the subject. The general interest which microscopy has attracted in Germany may be judged by the facilities which her universities furnish for its study. In the great London University a thorough acquaintance with microscopy has been recently demanded of candidates for graduation. Our own colleges are fast moving in the same direction. This new instrument and method of research are becoming known even to the masses. Enthusiastic popular meetings are held in its interest, and over all the land microscopial societies are springing into existence.

While news of the achievements of this wonderful instrument are wafted to us on every breeze, let us examine what it has already accomplished, what it is now doing, and what it is likely to do to advance the art of healing. The time has come when dentists can no longer confine their studies to the teeth and associate parts. Normal vital action, the problem of disease, of growth and repair, histological anatomy, and indeed histological physiology of the whole organism, are our direct province. Our art demands of us an exploration of all the phenomena of life, though our gathered resources are at last to be centured upon a territory but little more extensive than the oral cavity. Dentists and physicians begin to realize that the microscope can greatly aid them, and is actually necessary to an explanation of the mysteries of that vital action, which is a chief agent in the processes of healing. But they see also that it is something more than an aid to philosophical research; it is an every day necessity to the practitioner, and may lead to a much keener discrimination between similar and obscure forms of disease than can be made by the unaided senses.

This I take to be the attitude of the best minds in the two professions which have in charge the public health. Is it not then incumbent upon us to look to it that we do not fall behind those leading investigators who would open to us a new world of research and a new means of professional success? Let us inquire a little into the nature of the debt which medicine and dentistry owe to this most important of recent instruments of scientific research.

1st. To the microscope is due that great Cell-Doctrine, which is the basis of modern rational views of the phenomena of organic life. Without this, therapeutics in either medicine or dentistry could have had no sure foundation, since there could have been no true knowledge of the animal tissues and fluids either in health or disease. Our conception of the minute structure of the animal organism has been, therefore, literally wrested from the world of the unknown.

The science of histology, in its double aspect of anatomy and physiology, could have had no existence but for this instrument. For bodies below the five-hundreth of an inch in diameter are not perceived by the keenest natural eye, and the cell or organic unit is much smaller than this. These form-elements cannot therefore be seen, much less studied, until optical aid has expanded their area and rendered them visible. By an appropiiate magnifying power they may be seen, their form, arrangements, and external characters made out, and their internal structure, movements and forces ascertained.

The immense importance of these brilliant discoveries, which have been made in animal physiology through microscopic research, cannot be realized until the discoveries themselves are considered and the vital laws toward which they point understood. It may then be seen that the microscope has been to the physiologist the open door into a new world of strange forms and unsuspected forces. Entering this world the investigator observes phenomena, the bare existence of which he had never before suspected, and sets himself to study those vital laws, which would have been inconceivable previous to the introduction of the Cell-Doctrine into animal physiology by Schwann.

Through microscopic investigation a flood of light has been let in upon the constitution of every animal secretion and liquid. The blood was once regarded as fibrine and serum. There was absolutely no means of knowing that it is a transparent fluid, filled with billions of floating cells, both white and red, with the thousand interesting and significant facts that soon followed in the wake of this discovery. And that sublime spectacle which may be witnessed in the web of a frog's foot or a fish's tail, the vital stream sweeping impetuously along a multitude of anastomosing capillary tubes carrying white and red disks, could never have flashed its volume of instruction upon the brain of the physiologist but for the magnifying power of the lens. Previous to microscopic research, we knew little of the minute structure of enamel, little of cementum, little of dentine, little of the pulp, of secondary dentine or periosteum. We knew little of their relative life endowment, and little in fact of the minute structure of any organic substance. This meager knowledge of the normal states of tissue was of course a very poor foundation for any true theory of disease or rational method of treatment.

2d. The microscope has been equally active in bringing to light those deviations from normal structure, which are coexistent with disease. Long continued disturbances of function often result in minute changing of structure. These changes can in many cases be detected by a microscopal examination. Under the eyes of an army of competent investigators, such examinations have been instituted in almost every department of pathology. Though lacking that completeness and definiteness which time alone can give, these results are already of vast importance, as explaining many of the mysteries of pathological action, and as throwing greater ease and certainty into diagnosis.

As an instance of peculiar structure due to disease, the observations of Lebert may be mentioned. This distinguish observer describes certain characteristic corpuscular bodies in tubercle. Several other gentlemen have verified these observations, and have transplanted these tubercular cells to the skin of rabbits and guinea-pigs, which were afterward afflicted with tubercle, and in two or three months died. In like manner a specific living cell has been described on the mucus surfaces of the trachea in whooping cough patients, and has been repeatedly transferred to the respiratory surfaces of the rabbit, where catarrhal symptoms have ushered in the regular stages of a modified whooping-cough.

The peculiarities of situation in cancer have attracted much attention. Some observers assert and some deny that there is a specific cancer cell. It is not disputed however that a microscopial examination may determine the diagnosis, since in cancer there are chacteristic deviations from normal development and arrangement of cells.

Among a large number of eminent microscopial pathologists, who accept the germ theory of contagious diseases, the claims of Dr. Sansom may be mentioned. This gentleman maintains that the diseases of infection are due to microscopic atoms of vital matter or germs, which when "transmitted by physical intermedia to a healthy organism, soon infest the entire body." The same thing, substantially, is stated by Dr. Wm. Budd, when he maintains that "the specific cause of contagious fevers is a kind of living organism." Prof. Tyndall says that there are the "strongest grounds for holding all contagious matter to be particulate, and further that the particles are to all intents and purposes germs." Dr. Hollier's discovery of a distinct form of fungus in the blood of typhus fever patients, which he regards as the cause of the disease; and Dr. Saunderson's communication, made this winter to the Pathological Society of London, in which he maintains that bacteria in the blood are the cause of pyemia, have a bearing in the same direction.

Perhaps it is premature to consider this question settled, while an observer as distinguished as Dr. Beale stands opposed to it. Many of the facts are undeniable since they have been repeatedly verified by distant and independent observers; it is in their mode of explanation that we encounter differences of opinion. Dr. Austin Flint encourages the expectation that "microscopic researches in this direction may lead to developments of great importance in their bearing on our knowledge of the causation, the pathological character, and the treatment of many diseses."

It may be said that should germs or bacteria be proved to be effects or concomitant circumstances, either caused by the disease or made possible by the conditions brought in by the disease, and not primary agencies of morbid action they will yet hold an important place as a means of diagnosis. Thus, a great number of algæ and fungi have been described by different observers, some of which are characteristic, and which are therefore important diagnostic criteria of the disease which they accompany. A careful microscopcal examination in some of these cases will fix the diagnosis hours earlier than could have been done without this important aid.

Among skin diseases, Dr. Austin Flint describes five varieties, which are distiuguished by microscopic parasites that are characteristic, and which may be at once diagnosed by this peculiarity.

The discovery of the acarus scabei and the trichini spiralis have added to our knowledge of the diseases known as common itch and trichinosis. If symptoms are present which indicate the earlier stage of trichinosis, a microscopic examination of the pork last eaten may furnish corroborative evidence. Or, during a latter stage of the disease, the diagnosis may be rendered certain by harpooning a muscular fibre from the effected part and placing it under the lens. These and other facts indicate how important a part the microscope must play in our incoming era of medical investigation.

To finish the enumeration of such important diagnostic criteria as the microscope has revealed throughout the entire range of pathology, would be to write a book. This would be foreign to my present purpose, which is only to furnish a few hints to indicate the importance of the subject. Enough has already been said to show that the microscope is valuable beyond all danger of over-estimate to him who would promptly detect disease, and discriminate between its various forms.

The physician or dentist who would keep abreast with the times, do justice to his patients, and contribute as he may by accidental observations to the general fund of physiological and pathological knowledge, should make the microscope as familiar and constant a companion as the excavator or the lancet.

